# Effects of the α_1_-adrenoceptor agonist phenylephrine on SART stress-induced orthostatic hypotension in rats

**DOI:** 10.1186/1751-0759-4-13

**Published:** 2010-10-12

**Authors:** Yoshinori Funakami, Eiji Itoh, Taeko Hata, Tetsuyuki Wada, Seiji Ichida

**Affiliations:** 1 Kinki University School of Pharmacy, Kowakae 3-4-1, Higashi-Osaka, 577-8502, Japan

## Abstract

**Background:**

Specific alternation of rhythm in temperature (SART)-stressed rats, an animal model of autonomic imbalance, exhibit low blood pressure and tachycardia during consciousness and under anesthesia. In addition, these rats easily develop orthostatic hypotension (OH) as a response to postural manipulation. Hence, we studied the influence of the adrenalin α_1_-receptor agonist phenylephrine on stress-induced OH in SART-stressed rats and unstressed rats.

**Methods:**

Male Wistar rats weighing 250-300 g were used. Rats were fixed in the supine position under urethane anesthesia. Blood pressure was directly measured from the left common carotid artery and ECG was recorded simultaneously.

**Results:**

The maximum decrease in blood pressure and the area under the blood pressure-time curve were both large, while the %reflex was small in the SART-stressed rats compared with unstressed rats. In the SART-stressed rats, prolonged intravenous administration of phenylephrine reduced OH at a dose that barely affected unstressed rats.

**Conclusion:**

The results suggested that sympathetic dysfunction is a factor underlying SART stress-induced OH.

## Background

Autonomic dysfunction is the collapse of autonomic balance due to excessive stress. It is not considered to be a cause of orthostatic hypotension (OH) in the absence of abnormal physical findings [1]. In OH, cardiovascular sympathetic dysfunction tends to be a main causative factor. Various pharmacotherapeutic options are available to achieve functional improvement in such cases, especially, that using α_1_-adrenoceptor agonists [2].

Even when patients undergo various tests, OH diagnosis is usually difficult in the absence of abnormal findings and when blood pressure (BP) is maintained in the normal range. The utility of the head-up tilt (HUT) test is reported for neurally-mediated syncope, a condition associated with OH [3,4]. However, the angle of inclination, examination time, and the criteria for evaluation of the HUT test have been different in various reports, and there is no unified method for performing it yet [5]. Moreover, present methods are intended for humans, and there are few reports regarding the performance of the HUT test in experimental animals. SART-stressed rats, showing autonomic imbalance, is an experimental animal model in which OH can be naturally induced without using drugs.

In early spring and fall, climatic fluctuations including intense changes in the day-to-day temperature are encountered, because of which many people develop various illnesses. Specific alternation of rhythm in temperature (SART)-stressed rats represent an animal model that reflects the abovementioned clinical situation [6]. Based on the results from the tests conducted by Aschner and Mecholyl [7], these animals are accepted as an animal model of vagotonia-type dysautonomia and used as models for autonomic imbalance [7], hyperalgesia [8], and hypotension [9].

SART-stressed rats suffer from chronic hypotension and OH is easily induced in them [10]. They have a high heart rate (HR) and a high R voltage in ECG [6,11]. Hypotension and OH in such rats are improved by the administration of the M_2_-muscarinic receptor antagonist AF-DX116 [9,12]. The involvement of cardiac β_1_-adrenoceptors and vascular β_2_-adrenoceptors in OH has been suggested on the basis of experimental results after administration of various β-blockers [13].

In the present study, we studied regulation by sympathetic nerves using the HUT test in SART-stressed rats. We also investigated the effect of administering the selective α_1_-agonist phenylephrine on severe OH caused by the postural changes in these rats. Results were obtained as mean BP (MBP) measured automatically using electrocardiograms and BP-wave form recognition frequency change analysis software (Fluclet^®^). Furthermore, we examined phenylephrine-induced vasoconstriction of the isolated thoracic aorta, carotid and mesenteric arteries using the Magnus method to investigate blood circulatory changes on rising up from a supine posture.

## Material and methods

### Experimental animals and procedure for SART stress loading

#### Experimental animals

Male Wistar rats (Japan SLC, Inc., Hamamatsu) weighing 250-300 g at the start of the study were used in accordance with ethical procedures following the guidelines for the care and use of laboratory animals issued by the Japanese government and The Japanese Pharmacological Society. The animals were housed in groups of three in a wire-net cage (38 × 25 × 17 cm) placed in a temperature- and light-controlled room (24 ± 1°C with a 12-h light-dark cycle; lights on at 08:00, off at 20:00), and were provide access to a standard diet (MF; Oriental Yeast, Tokyo) and tap water *ad libitum*.

#### Procedure for SART stress loading

According to procedures reported previously [14], three rats per group were alternately transferred to two cages, one of which was placed in a room at 24°C and the other in a room at -3°C, every hour from 09:00 to l6:00 and then housed in a cage at -3°C from l6:00 to 09:00 the following morning. This procedure was repeated for 6-8 days until 11:00 on the day of the experiment. The stressed rats were kept at room temperature (24°C) for at least 30 min before the experiment to avoid the direct influence of the cold environment. Unstressed rats were housed in a room at 24°C all day because no influence was observed when mice were moved according to the same schedule as the SART-stressed rats into two separate cages maintained in a room at 24°C [14].

### Drugs

Urethane (SIGMA), heparin (Shimizu Pharmaceutical), and the selective α_1_-agonist, phenylephrine hydrochloride (SIGMA) were used. The above drugs were dissolved in 0.9% physiological saline at use, and concentrations were prepared so that doses were 0.1 mL/100 g when those were administered to rats. Phenylephrine was continuously injected into the left femoral vein at a rate of 1 μg/kg/min.

### Measurement of MBP and HR

Urethane (1.2 g/kg, i.p.)-anesthetized rats were restrained on a board in a supine position according to our previous study [13]. In that study, BP, HR and tilting parameters obtained from the carotid artery were reported to be similar to those obtained from the femoral artery, but the differences in the parameters between unstressed and SART-stressed rats were larger and clearer in the data from carotid artery. Therefore, data from the carotid artery were used in this study. A polyethylene cannula PE50 (Clay-Adams, Division of Becton Dickinson and Company, Parsippany, NJ, U.S.A.) was inserted into the left common carotid artery. The other end of the tube was connected to a pressure transducer (DX312, Nihon Kohden, Tokyo). BP was measured and the electrocardiogram was recorded using Fluclet (Fluclet^® ^Jr.2: Dainippon Pharmaceutical Co., Ltd., Osaka) through strain amplifiers (AS 1202, NEC SanEi, Tokyo). Electrocardiographic complexes were recorded through lead II, and HR (beats/min) was calculated on the basis of the R-R interval. To prevent blood coagulation in the cannula, a heparin 10 IU/mL solution was injected at a speed of 0.38 mL/h. The board was kept warm at about 37°C to maintain the body temperature of the rats. If the experiment extended for many hours, a small dose of urethane was added as required (1/10 of the initial dose, i.p.)

### HUT test

The HUT test, or a stimulation of postural change, was performed according to procedures reported previously [13]. After BP and HR were stabilized, they were measured and recorded for 2 min in a supine position before the HUT test was initiated. Postural change was performed by rapidly lifting the head portion of the board to a 60° head-up position from the horizontal position. This inclination (standing position) was maintained for 4 min and the rats were then returned to the original, horizontal position. Measurements were performed immediately after the changes of position and at 5 s, 10 s, 15 s, 30 s, 1 min, 1.5 min and 2 min and every minute thereafter until 8 min after the change of position (4 min after returning to the horizontal position). Similar to our previous report, three parameters (tilting indices) were used for indicating the degree of OH [13]. These parameters were derived from BP change-curves calculated on the basis of the pressure values measured above. In short, the tilting indices were as follows: (1) the maximum decrease (MD) in BP caused by the HUT, (2) %reflex obtained from the change in position (the ratio of the maximum increase within 2 min after raising the board from the lowest level of the decreased BP to MD), and (3) the area under the curve (AUC), enclosed between the baseline and the recovery curve of BP from 0 to 4 min (mmHg· min). MBP was used for calculating all these indices.

Drug administration began after the control HUT test, and the effects of drugs on the HUT test were evaluated 4 times at 15, 45, 75 and 105 min after the start of drug administration.

### Blood vessel preparation

Rats were anesthetized with ether. The thoracic aorta, carotid, and mesenteric arteries were excised and placed in ice-cold oxygenated (95% O_2_-5% CO_2_) and modified Krebs-Henseleit solution (KHS) [in mM: NaCl, 154; KCl, 1.7; MgSO_4_·7H_2_O, 1.2; KH_2_PO_4_, 2.5; CaCl_2_, 1.9; NaHCO_3 _, 25; Glucose, 12 (Wako Pure Chemicals Inc, Osaka)]. The vessels were dissected and cut into 2.0-mm segments (the thoracic aorta and carotid arteries) and 1.5-mm segments (second-order branches in the mesenteric arteries).

### Measurement of isometric tension

Optimal tension was determined by subjecting arterial segments to different resting tensions (the carotid artery: 1.0 g, the thoracic aorta: 600 mg and the mesenteric artery: 100 mg). The chambers were replaced with fresh KHS every 15 min for 60 min, and the segments were stretched to optimal tension. Mesenteric artery segments were mounted isometrically on a pen recorder (TYPE3056, Yokogawa Electric Corporation., Tokyo) using a micro-tissue organ bus (MTOB-1, LABO SUPPORT Co. Ltd., Suita) for measuring the generated force. The chambers were kept at 37°C and bubbled continuously with 95% O_2 _- 5% CO_2 _in KHS.

### Statistical analyses

Experimental data were expressed as mean ± standard error of the mean and statistically analyzed by an unpaired Student's *t*-test for data from two groups, and by one-way or two-way analysis of variance (ANOVA) and Tukey's test for data from multiple groups. Significance was set at P < 0.05.

## Results

### Influence of SART stress on MBP and HR during the HUT test

**Figure 1:**
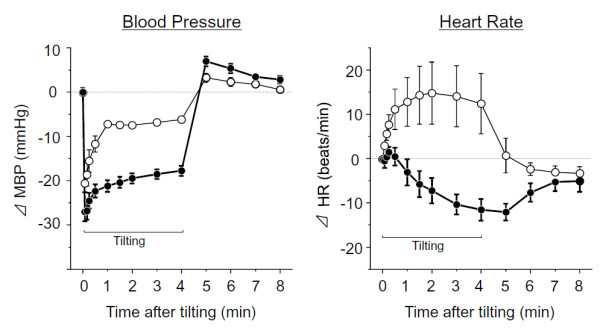
**Influence of SART stress on MBP and HR at tilting in anesthetized rats**. Data show the mean ± S.E.M. from 8 unstressed rats (open circle) and 8 SART-stressed rats (closed circle).

Time-related changes in MBP and HR with the HUT test are shown in Figure 1. In unstressed rats, BP maximally decreased immediately after tilting, and increased shortly thereafter, recovering to within -10 mmHg from the baseline. In contrast, in the SART-stressed rats, (i) the maximum BP drop observed just after tilting was larger than that for unstressed rats, (ii) the recovery from the immediate drop in BP was slow and rose only to about -20 mmHg from the baseline, and (iii) BP did not return to the level seen in unstressed rats.

HR increased immediately after tilting in unstressed rats, and returned to the baseline after the HUT test ended. In contrast, in SART-stressed rats, HR did not increase after tilting, but rather decreased and remained at the decreased level for several min even after returning back to supine position. These changes which were automatically measured and recorded by Fluclet as reported previously [13].

**Figure 2:**
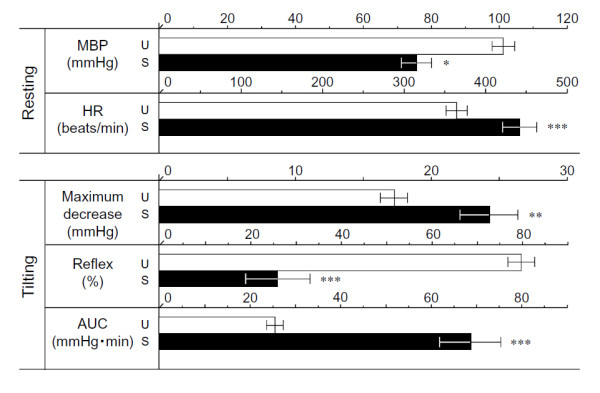
**Influence of SART stress on tilting parameters in anesthetized rats**. Data show the mean ± S.E.M. from 7 unstressed rats (U, open column) and 9 SART-stressed rats (S, closed column). *P < 0.05, **P < 0.01, ***P < 0.001 vs respective unstressed group (*t*-test).

Figure 2 shows resting MBP, HR, MD, %reflex and AUC with the HUT test. Resting MBP measured in the supine position was 100.5 ± 3.4 mmHg in unstressed rats, and 75.3 ± 4.5 mmHg in SART-stressed rats. HR of SART-stressed rats was 439.0 ± 20.9 beats/min, which was significantly greater than the value of 365.9 ± 8.4 beats/min in unstressed rats.

The maximal decrease in MBP and AUC due to tilting in the stressed rats was 24.3 ± 2.1 mmHg and 68.9 ± 6.8 mmHg·min, respectively, significantly higher than the values for unstressed rats (l7.3 ± 1.0 mmHg and 25.5 ± 1.9 mmHg·min, respectively). The other parameter, %reflex in SART-stressed rats, was 25.9 ± 7.0%, which was smaller than that (79.2 ± 3.0%) in unstressed rats.

### Effects of the selective adrenaline α_1_-agonist phenylephrine

#### 1) Changes in MBP and HR by the HUT test

**Figure 3:**
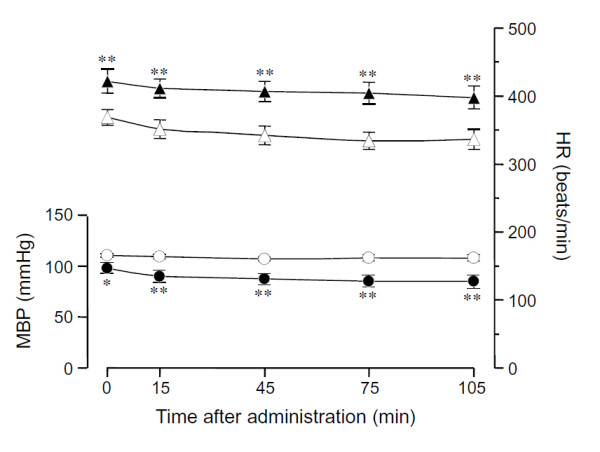
**Effects of phenylephrine on time-related changes in resting MBP and HR**. Circle; MBP and triangle; HR. Open circle and triangle; Unstressed rats and closed circle and triangle: SART-stressed rats. Data show the mean ± S.E.M. of 7 unstressed and 9 SART-stressed rats. Phenylephrine 1 mg/kg/min, was continuously administrated by i.v.-infusion. Values at 0 min are control values before administration of phenylephrine.

Figure 3 shows the effects of phenylephrine on resting MBP and HR. Changes in resting MBP and HR after administration of phenylephrine were observed from 15 min to 105 min.

**Figure 4:**
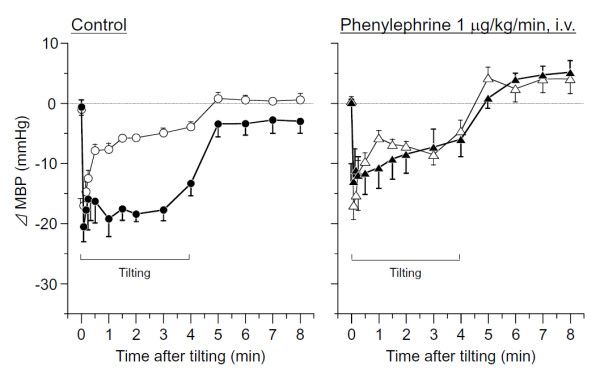
**Effects of phenylephrine on time-related changes in MBP caused by tilting in anesthetized rats**. Open circle and triangle; Unstressed rats and closed circle and triangle: SART-stressed rats. Data show the mean ± S.E.M. of 7 unstressed and 9 SART-stressed rats. Phenylephrine, 1 mg/kg/min, i.v., was continuously administrated by i.v.-infusion from 15 min before tilting.

The influence of phenylephrine on MBP is shown in Figure 4. In unstressed rats, phenylephrine did not change MBP. In SART-stressed rats, the sudden BP drop observed in controls just after tilting was inhibited or decreased by phenylephrine, and the change in BP was similar to unstressed rats. Continuous infusion of physiological saline did not change MBP and HR. Data before drug administration (baseline data) were used as the control values of the HUT test.

**Figure 5:**
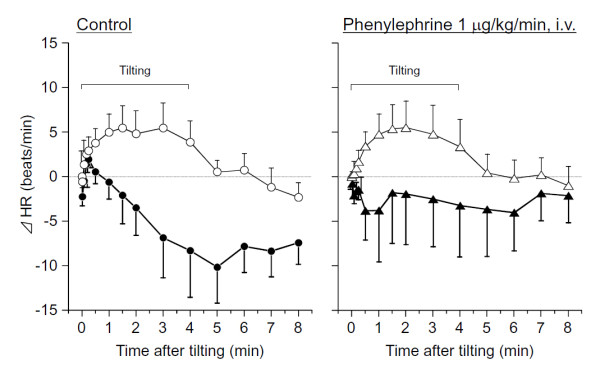
**Effects of phenylephrine on time-related changes in HR caused by tilting in anesthetized rats**. Open circle and triangle; Unstressed rats and closed circle and triangle: SART-stressed rats. Data show the mean ± S.E.M. of 7 unstressed and 9 SART-stressed rats. Phenylephrine 1 mg/kg/min, was continuously administrated by i.v.-infusion from 15 min before tilting.

Figure 5 shows the effects of phenylephrine on the change in HR. Effects of phenylephrine were not observed in unstressed rats. However, in SART-stressed rats, the bradycardia after tilting tended to be inhibited by phenylephrine. The change after administration of phenylephrine was observed from 15 min to 105 min.

#### 2) OH indices: MD, %reflex, and AUC

**Figure 6:**
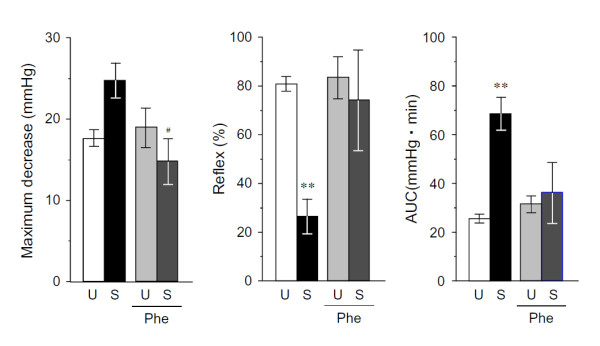
**Effects of phenylephrine on orthostatic hypotension parameters in tilting in rats**. Phe: Phenylephrine, 1 mg/kg/min, i.v.. Data show the mean ± S.E.M. from 7 unstressed rats (U) and 9 SART-stressed rats (S). **P < 0.01 vs respective unstressed control group. ^#^P < 0.05 vs respective control group (Tukey's test).

Figure 6 shows results at 15 min after phenylephrine administration. In unstressed rats, MD and %reflex before phenylephrine administration were 17.3 ± 1.0 mmHg and 79.2 ± 3.0%, respectively, and hardly changed after administration of the agent. In SART-stressed rats, MD and %reflex before phenylephrine administration were 24.3 ± 2.1 mmHg and 25.9 ± 7.0%, respectively, which were significantly larger and smaller than the values for the unstressed group. Following administration of phenylephrine, MD decreased to 14.7 ± 2.9 mmHg and %reflex greatly increased to 72.6 ± 20.2%, demonstrating a marked improvement. Percentage reflex of the SART-stressed rats as measured with the HUT test was greatly increased from the baseline state by phenylephrine, and the value returned almost to the baseline level in unstressed rats.

Regarding AUC, there was hardly any phenylephrine-induced change in the unstressed group. The AUC in the SART-stressed group following administration of phenylephrine, considerably decreased to 35.3 ± 12.1 mmHg·min from the unstressed value of 68.9 ± 6.8, demonstrating marked improvement. In this way aggravation of OH by SART stress was ameliorated by phenylephrine at a dose that was ineffective in unstressed rats.

#### 3) Vasoconstriction in the thoracic aorta, carotid and mesenteric arteries

**Figure 7:**
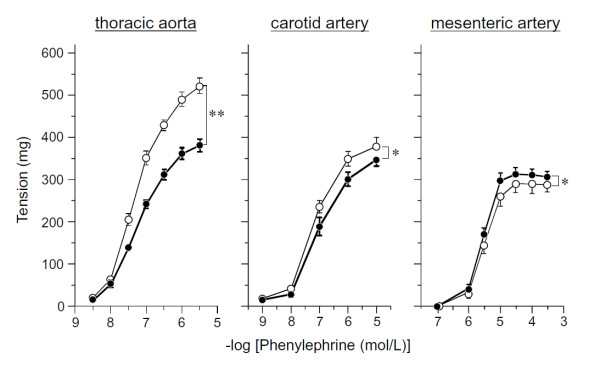
**Influence of SART Stress on phenylephrine-induced contraction in the isolated various arteries of rat**. Data show the mean with S.E.M. of 7 to 16 preparations. Open circle; Unstressed rats, closed circle; SART-stressed rats. *P < 0.05, **P < 0.01 vs the unstressed rats (Two-way ANOVA).

Figure 7 shows vasoconstriction caused by cumulative addition of phenylephrine to vascular smooth muscle *in vitro*. The reactivity of the thoracic aorta decreased significantly in the SART-stressed group in comparison with the unstressed group. Contractile reduction was also observed in the common carotid artery in SART-stressed rats. On the other hand, in the mesenteric arteries, the vasoconstrictor response increased significantly in the SART-stressed group compared with the unstressed group.

## Discussion

Classifications of OH vary. Some research on the subclassification of instantaneous OH, postural tachycardia syndrome, neurally-mediated syncope, and delayed OH has been conducted [15]. However, if there are symptoms of an underlying condition, selective α_1_-agonists are used for treatment of OH, and such therapy improves sympathetic nerve function [2].

We have already reported that in SART-stressed rats, HUT test-induced OH develops more frequently than in unstressed rats, and can be classified into four types on the basis of BP fluctuations that resemble the four types of OH observed in man as noted above [10]. In the present study, stress-induced OH in rats was improved by the selective α_1_-agonist phenylephrine. These symptoms were previously reported to be improved by a selective M_2_-receptor antagonist, AF-DX 116 [9,12]. M_2_-receptors contribute to persistent hypotension but M_1_-receptors do not. Increased sensitivity of M_2_-receptors located on sympathetic nerve endings in the heart and decreased sensitivity of myocardial M_2_-receptors have been suggested as probable etiologies for the phenomenon [9]. Involvement of M_2 _receptors in the manifestation of OH in SART-stressed rats has been demonstrated [12]. We applied the isoproterenol-treated HUT test, which is used for diagnosis of neurally-mediated syncope, on SART-stressed rats. Effects of β-blockers on OH show involvement of a cardiac β_1_-receptors and vascular β_2_-receptors. When expression of OH is strong, an accommodative disorder of the sympathetic nervous system is thought to be involved [13]. The primary cause of OH is considered to be autonomic dysfunction but secondary causes include dehydration, malnutrition (vitamin deficiency) or hypometabolism secondarily. SART-stressed rats do not show abnormalities in the electrolytes (Na^+^, K^+^), GOT (AST) and GPT (ALT) in serum [16], and dehydration. SART-stressed animals have a voracious appetite [17]. It is thought that dehydration and a suppression of metabolism do not occur in SART-stressed rats, although further study is needed regarding vitamins.

SART-stressed rats are associated with hypotension and tachycardia [6,9,12,13], and severe OH is easily induced by postural manipulation [10]. Compared with unstressed rats, SART-stressed rats show large MD and smaller %reflex, but show no compensatory tachycardia caused by the fall in BP. MD is the maximal fall in MBP with the HUT test and is used diagnosing OH. Percentage reflex is a value reflecting a compensation of sympathetic function that normalizes BP after the decrease caused by the HUT test. AUC reflects MD and %reflex, and can indicate the intensity of OH. SART-stressed baseline tachycardia, and reflex systems regulating HR are considered to be inhibited.

To investigate improvement effects on OH, the dose of phenylephrine without influence on resting MBP in both unstressed and SART-stressed rats was determined as 1 μg/kg/min. Continual infusion of this dose gives similar OH indices that indicate improvement effects from 15 to 105 min and therefore values at 15 min were used as representative figures.

Phenylephrine improved OH in SART-stressed rats and showed effects on MD, %reflex, and AUC after the HUT test. Because MD decreased in SART-stressed rats, a decrease in sympathetic tone and peripheral vessel vasoconstriction was speculated. During the continuous infusion of phenylephrine, the improvements in sympathetic tone and peripheral vessel vasoconstriction were similar to those from continuous infusion of the adrenalin β-receptor agonist isoproterenol.

It is known that vascular resistance in peripheral arteries such as mesenteric arteries is strongly related to BP [18-23]. OH occurs after a change to the standing position from the resting position: because of a combination of three factors, arteriolar hyposystolic failure in the resistance vessels, decrease in hemoperfusion volume and cardiac output by weakness of the inferior limb muscles and abdominal muscles, and reduction of venoconstriction. In our study, the constriction reaction of various vascular smooth muscles to phenylephrine in SART-stressed rats decreased in the thoracic aorta and carotid arteries and increased in the mesenteric arteries. *In vivo*, after phenylephrine infusion, the contraction of the mesenteric arteries in SART-stressed rats decreased the blood pooled in the vascular bed. The constrictive balance of the carotid and mesenteric arteries circulated blood to the brain more efficiently. It is thought that these results greatly contribute to improvement in SART stress-induced OH. It is already known that blood flow in SART-stressed rats decreases in the carotid artery but increases in the abdominal and mesenteric arteries [24], and plasma noradrenaline (NA) levels are several times higher than those in unstressed rats [25]. The plasma NA level increased to several fold in people who were changed to a standing position. In other words, in SART-stressed rats, blood flow in the carotid artery and vasoconstriction of the thoracic aorta remarkably decreased along with the sympathetic tone during the HUT test. Furthermore, it is thought that OH is strongly manifested, and a transient cerebral ischemia is caused by an increase in blood flow to the mesenteric arteries during the HUT test.

Today, selective α_1_-agonists increase BP by predominantly causing vascular smooth muscle constriction, and are the therapeutic drugs for various types of hypotension including OH. Many of these drugs cause tachycardia to increase BP instantly. This can cause discomfort and a stressful sensation. In pharmacotherapy of OH, it is important to assist the sympathetic nerve function to develop a substitutive reflection caused by standing.

Stress and the autonomic nervous system strongly influence the development of hypotension and OH. Sympathetic hypoactivity is considered a cause of these diseases, and most pharmacotherapeutic options for these diseases target the sympathetic nervous system. However, BP is regulated not only by the sympathetic nervous system but also by the parasympathetic nervous system. We have already reported that the parasympathetic nervous system has an important role in the pathogenesis of OH (12). Our understanding of the role played by the parasympathetic nerve in OH is still poor, but we are paying more attention to this area and plan to study it further.

## Competing interests

The authors declare that they have no competing interests.

## Authors' contributions

YF was the main investigator, contributed to the data collection and wrote the first draft of the manuscript. TH supervised the study, analyzed the data and wrote the final draft of the manuscript. EI and TW and SI supervised the study. All authors contributed to the preparation of the article and approved the final manuscript.
